# Targeting HTLV-I latency in Adult T-cell Leukemia/Lymphoma

**DOI:** 10.1186/1742-4690-8-S1-A48

**Published:** 2011-06-06

**Authors:** Juan C Ramos, Ngoc Toomey, Luis Diaz, Phillip Ruiz, Glen Barber, William Harrington

**Affiliations:** 1 University of Miami, Miami, FL, 33136, USA

## 

Adult T-cell leukemia/lymphoma (ATLL) is highly chemotherapy resistant. The combination of AZT and interferon (IFN) is a first line treatment option for the leukemic forms of ATLL. We and others have demonstrated AZT/IFN can effectively suppress ATLL long-term; however, these drugs fail to eradicate malignant ATLL clones. At our institution we have recently established a clinical trial for ATLL using AZT/IFN in combination with the inexpensive histone deacelytase (HDAC) inhibitor valproic acid (VPA) during the maintenance treatment phase. Histone acetylation can result in HTLV-I promoter activation and viral transcription. We hypothesized that HDAC inhibitors would re-activate latent HTLV-I in ATLL cells harboring intact provirus and help eliminate residual disease after cytoreductive treatment. We have exciting preliminary data which suggest we can achieve this. So far, we have enrolled 13 subjects with acute-type ATLL in our study. We observed a serial decrease in clonal ATLL disease followed by molecular clearance by multiplex PCR in one subject after VPA treatment. We had not seen such effect previously in long-term responders treated with AZT/IFN alone. Using fresh ATLL cells from this subject we augmented HTLV-I expression and induced cell death ex vivo after treatment with the newer HDAC inhibitor vorinostat. We are currently testing other newly available HDAC inhibitors in our pre-clinical models. The dual anti-neoplastic and viral inducing roles of HDAC inhibitors can be exploited in the treatment of ATLL. This exciting approach may help advance the cure for this disease. We will present our interim clinical trial results at the conference.

